# “I never thought to bring them up to the doctor”: examining symptom appraisal, help-seeking behaviors, and diagnostic delays among young adults with cancer

**DOI:** 10.1007/s10552-026-02231-3

**Published:** 2026-08-22

**Authors:** Natasha C. Allard, Elizabeth G. Bouchard, Jennifer S. Ford, Thomas Hugh Feeley, Denise Rokitka, Heather Orom

**Affiliations:** 1 Department of Community Health and Health Behavior, School of Public Health and Health Professions, University at Buffalo, Kimball Tower, Buffalo, NY 14214 USA; 2 Department of Cancer Prevention and Control, Roswell Park Comprehensive Cancer Center, Elm and Carlton Streets, Buffalo, NY USA; 3 Department of Psychology, Hunter College and The Graduate Center, City University of New York, 695 Park Avenue, HN 611, NY, NY USA; 4 Department of Communication, College of Arts and Sciences, University at Buffalo, Baldy Hall, Buffalo, NY 14260 USA; 5 Roswell Park Comprehensive Cancer Center, Department of Pediatrics, Elm and Carlton, Buffalo, NY 14263 USA

**Keywords:** Young adult cancer, Diagnostic delays, Symptom appraisal, Help-seeking behavior, Communication interventions, Qualitative research

## Abstract

**Purpose:**

Cancer incidence is rising among young adults (20–39 years), and it is one of the top five causes of death for this age group. Young adults often experience longer delays in diagnosis than children and older adults, which negatively impacts survival and quality of life. Communication interventions might help reduce these delays, but understanding the factors driving delays is crucial for developing effective strategies.

**Methods:**

We conducted semi-structured interviews with 30 young adults diagnosed with cancer to explore factors contributing to delays across three intervals: symptom appraisal, help-seeking, and diagnostic. Interviews were conducted in January and February 2023 and analyzed using template analysis by three independent coders.

**Results:**

Most young adults misattributed symptoms to stress, lifestyle, or minor issues, and thus delayed seeking medical evaluation. Many used online information to inform care-seeking decisions but encountered content that minimized symptom severity or offered unclear guidance. Upon seeking care, several young adults had to self-advocate for diagnostic testing after providers initially dismissed their concerns.

**Conclusion:**

These patterns highlight potentially modifiable targets for future communication interventions. Strategies to improve young adults’ awareness of potential cancer symptoms, address misconceptions about cancer risk, and promote timely care-seeking are warranted. Efforts should also focus on improving the quality of online symptom information and equipping young adults with tools to communicate more effectively with providers. While systemic changes are also needed, individual-level communication campaigns could help shorten time to diagnosis and improve outcomes for young adults with cancer.

## Introduction

Cancer is the fifth leading cause of death for young adults (20–39 years) in the United States [1–3]. Young adults experience longer delays in diagnosis than children and older adults [4–11], and these delays are associated with diminished quality of life, increased burden of co-morbidities, and poorer survival outcomes [1, 10, 12–16]. Communication interventions hold potential to reduce these delays, but a clear understanding of the factors driving delays is essential for developing effective strategies. Due to limited routine screening for asymptomatic cancer in young people, most young adults are diagnosed only after presenting to a medical provider with symptoms [4, 17]. This reliance on individuals to recognize symptoms and seek care highlights a critical opportunity to promote earlier help-seeking through targeted communication. However, little is known about the psychological, behavioral, and structural factors that influence how young adults interpret symptoms and navigate the diagnostic process. To address this gap, we used the Model of Pathways to Treatment [18, 19] to examine cancer diagnosis delays among young adults and identify targets for earlier diagnosis and improved outcomes.

### Research about young adult cancer diagnosis delays

Although research on diagnostic delays in this age group is limited, several potential barriers to timely diagnosis have been identified, including structural, provider-level, and individual-level factors. Among structural determinants, living in a rural (versus metro) location and being uninsured or underinsured are associated with young adult delays [20–30]. Medical providers’ lack of awareness that symptoms could be cancer, or attribution of symptoms to lifestyle causes, may also lengthen diagnosis timelines [31, 32].

Individual-level factors can contribute to prolonged diagnostic journeys; however, most research had been conducted outside the US where healthcare access conditions differ. For example, a study in the United Kingdom (UK) found that 27% of adolescents and young adults who were diagnosed with cancer waited more than one month to seek help for their symptoms [6]. In several qualitative studies, young adults with cancer believed their pre-diagnosis lack of cancer symptom knowledge and feeling of invincibility contributed to not seeking medical care sooner [31, 33–35]. Additional UK-based research identified practical and emotional barriers to timely care-seeking, including challenges of missing school or work for appointments, competing priorities with family, and worries or fears associated with talking to a doctor [36–38]. US-based research on care-seeking and diagnosis delay in this population remains scarce, though one study examining a related construct, time to treatment, found that young adults with Hodgkin lymphoma experienced longer delays than adolescents, attributed in part to the competing work and family responsibilities characteristic of this life stage [39].

While these findings provide useful insights into young adult behavior in other countries and contexts, and some US-based research has explored how older adults make decisions to seek care, little is known about how these processes unfold specifically for young adults in the US. This gap warrants further investigation given the key developmental, psychological, and situational differences between young adults and older populations. Moreover, young adults aged 20–39 represent a distinct subgroup within the broader adolescent and young adult (AYA) population (ages 15–39), as they differ from adolescents in cancer type distribution and in their need to navigate adult medical systems [11]. Distinct characteristics of young adulthood, such as emerging autonomy [25], growing education, work and family responsibilities [40], and increased reliance on online health information [41], may influence how symptoms are interpreted and when care is sought.

### The model of pathways to treatment

The Model of Pathways to Treatment [18, 19] divides the pathway to diagnosis into four time periods. The appraisal interval begins when an individual detects a bodily change (e.g., vision changes, blood in stool, etc.) and involves assessing whether that change represents a normal fluctuation or an abnormal symptom, sometimes drawing on non-clinical sources such as online searches or conversations with others. The interval ends when the individual perceives the symptom as warranting consultation with a healthcare provider, though some may dismiss symptoms as nonserious or attempt to self-manage them before reaching that point. The help-seeking interval begins when an individual perceives a need for consultation with a healthcare provider and involves making decisions about contacting a provider to discuss symptoms. Challenges such as lack of insurance, inability to miss work, or absence of a routine provider may prolong this interval or motivate a return to symptom self-appraisal. The diagnostic interval begins at the point of first discussion with a healthcare provider and continues until a formal, accurate disease diagnosis is made. This interval encompasses all provider encounters, referrals, misdiagnoses, and provider-recommended symptom management strategies that may occur before a diagnosis is reached. The final interval, pre-treatment, is outside the scope of diagnosis delay research.

The Model of Pathways to Treatment is well suited to guide the present analysis because it outlines general events and stages that can be applied to any disease or population and has been well established for studying cancer diagnosis delays [42–46]. The model’s intervals and processes are supported with empirical findings. A UK study of 7,543 adults used the Model of Pathways to Treatment to help identify several significant barriers to medical care seeking for potential cancer symptoms during COVID-19 [47]. Another study used the model to identify predictors of delayed breast cancer diagnosis and treatment in South African women [42]. A nationally representative assessment of US adults’ reasons for avoiding medical care found multiple barriers consistent with processes and factors outlined in the Model of Pathways to Treatment [48].

### Study aims

We asked three research questions about delays in time to diagnosis (i.e., the time from detecting a bodily change to disease diagnosis) for young adult cancer survivors:

1. What prolongs the appraisal interval? Specifically, what appraisal and self-management processes and determining disease, patient, and health care system factors contribute to delays between noticing bodily changes and perceiving the need to consult a healthcare provider?
2. What prolongs the help-seeking interval? Specifically, what decision-making processes and determining disease, patient, and health care system factors contribute to delay between perceiving the need to consult a healthcare provider and consulting a healthcare provider for the first time?
3. What prolongs the diagnostic interval? Specifically, what health care experiences and determining disease, patient, and health care system factors contribute to delay between first healthcare provider consultation and formal cancer diagnosis?

Understanding young adult experiences within each of these three intervals will highlight potential opportunities for shortening the total time to diagnosis duration through communication interventions.

## Methods

### Participants and sampling

We conducted semi-structured interviews in January and February, 2023 with individuals who were diagnosed with any type of cancer between the ages of 20 and 39 years (*n* = 30). Although adolescents and young adults have often been studied as one age group (typically defined as 15–39 years old), combining the full age range into one group can mask important heterogeneity and limit comparison to other studies. We limited our study to the young adult age range of 20–39 years, consistent with the cut-off used in previous young adult cancer studies [11, 49] and supported by findings from the Adolescent and Young Adult Oncology Progress Review Group [50] that document meaningful differences between adolescents and young adults within the broader AYA population.

This study, including all procedures and materials, was approved by the University at Buffalo Institutional Review Board. Participants were recruited via ResearchMatch (26.7%, *n* = 8), an online volunteer registry for health-related studies [51], and through recruitment posts in cancer-related social media groups (73.3%, *n* = 22). Potential participants completed a REDCap screening survey to determine their eligibility. Eligibility criteria included diagnosed with cancer between 20 and 39 years old, currently between 20 and 45 years old, resided in the United States both at the time of the study and at the time of diagnosis, and experienced abnormal physical changes/symptoms before cancer diagnosis. The current age upper limit was selected pragmatically to maximize recruitment of a population that can be difficult to engage in research, while ensuring that participants remained close enough to young adulthood that their reflections on pre-diagnosis experiences would be grounded in the developmental, psychological, and situational context characteristic of that life stage. We purposively sampled only young adults who recalled experiencing symptoms because those who were diagnosed through routine bloodwork or screenings would not have experienced the appraisal or help-seeking processes central to our study aims and the goal of informing communication interventions. Importantly, in the screening survey, we did not ask if the symptom was the reason they sought medical care, simply if, in hindsight, they recall experiencing physical body changes.

### Procedure

The lead author (NCA) conducted semi-structured interviews with each participant. All participants provided informed consent. Each interview began with a broad question asking the participant to describe what happened in the time from the first physical change they noticed to the time they received a formal diagnosis. Follow-up questions asked about specific factors and processes impacting their appraisal of symptoms, decision to seek help for those symptoms, and healthcare encounters leading to diagnosis. Interviews lasted approximately 60 min and took place on Zoom. All interviews were video recorded and transcribed verbatim.

### Data analysis

Three independent coders (NCA, CB, LK) used template analysis [52, 53] to code each interview transcript. NCA developed an initial a priori codebook based on the study aims, interview guide, and existing research, which was refined through preliminary coding of four transcripts. The three coders then applied the codebook to six transcripts, met to resolve discrepancies, and finalized the codebook. The final codebook was applied to all transcripts by two coders each (NCA coded all 30 transcripts; CB and LK coded 15 each).

## Results

### Participant characteristics

Sample characteristics (*n* = 30) are presented in Table 1. The mean age of participants at the time of interview was 32.3 (SD = 6.0) years, and the mean age at diagnosis was 28.5 years (SD = 5.3). Most participants were non-Hispanic White (73.3%, *n* = 22) and female (56.7%, *n* = 17. At the time of diagnosis, 40.0% (*n* = 12) of participants were attending college or graduate school and 73.3% (*n* = 22) had a full-time job. Participants were diagnosed with 12 types of cancer, with more than one-third were diagnosed with a form of leukemia (36.7%, *n* = 11). Years since diagnosis ranged from less than one year to 17 years, with a mean of 3.8 years (SD = 3.71) and a median of 2 years.

**Table 1:** Sample characteristics (time of diagnosis)

	*n* or Mean	% or SD
Current age	32.3	6.03
Age at diagnosis	28.5	5.25
Years since diagnosis	3.8	3.71
Gender		
Female	17	56.7
Male	10	33.3
Non-Binary	3	10.0
Race/ethnicity		
Non-Hispanic White	22	73.3
Non-Hispanic Black	2	6.7
Hispanic	4	13.3
Asian	2	6.7
Marital status		
Single/Divorced/Widowed	17	56.7
Married/Cohabitating	13	43.3
Education		
> = College graduate	23	76.7
Some College/Post HS Training	6	20.0
HS Graduate	1	3.3
Geographic type		
Suburban	15	50.0
Urban	13	43.3
Rural	2	6.7
Income		
< $25 K	5	16.7
$25 K-49,999	4	13.3
$50 K-74,999	7	23.3
$75 K-99,999	8	26.7
$100 K +	6	20.0
Has children	6	20.0
In college	2	6.7
Working full-time	22	73.3
Has health insurance	29	96.7
Cancer type		
Leukemia	11	36.7
Brain	4	13.3
Breast	3	10
Lymphoma	2	6.7
Testicular	2	6.7
Thyroid	2	6.7
Ovarian	1	3
Colorectal	1	3
Cervical	1	3
Uterine	1	3
Melanoma	1	3
Sarcoma	1	3

## Research question #1: What prolongs the appraisal interval (i.e., time from noticing bodily change to perceiving a need for consultation with a healthcare provider)?

*Theme 1: Most young adults attributed ongoing but vague symptoms to mental and physical stress stemming from work, school, major life changes, or their unhealthy behaviors.* Nearly all young adults waited more than one month (usually several months, sometimes even years) to seek care after symptom awareness: “I had bleeding after sex and bleeding between menstrual cycles for a year [before I told my doctor].” *[Participant 17, diagnosed at 35, cervical cancer]* The most common reason for this delay was believing symptoms were caused by stressors; many perceived low urgency for consulting a healthcare provider because they assumed that symptoms would stop when their stress abated, life ‘calmed down’, or they changed their behavior: “It was like an hour commute every day, back and forth. And it was like, a lot of stress so we kind of chalked up symptoms to that for a long time.” *[Participant 14, diagnosed at 24, brain cancer]* A few participants recalled the exact time span or event in which they expected symptoms to dissipate, for example: “I had been experiencing fatigue for several months and kind of ignored it. I had four months to go until graduation and I figured I could just push through.” *[Participant 1, diagnosed at 35, leukemia*] The stressors that participants described were common for young adults, including starting college, beginning a new job, moving homes, marriage, financial autonomy, and growing a family.

Several participants believed it was expected for stress to cause physical issues: “But I remembered that during orientation they told us that freshmen year is the tiredest you will ever be.” *[Participant 5, diagnosed at 20, sarcoma]* Some participants attributed symptoms to frequent alcohol consumption, late night partying and studying, low water intake, and lack of physical activity:

I was just waking up super drenched in sweat, like, more than is appropriate. I was really, really, really tired a lot. But I was also at an age where I was drinking … ‘Oh, maybe I’m just, like, living too hard.’* [Participant 12, diagnosed at 24, leukemia]*

*Theme 2: Some participants dismissed symptoms as nonserious based on their perceived expectations of “normal” physical issues occurring to people like themselves.* A few participants living in colder climates (i.e., Northeast and Midwest states) did not perceive a need for healthcare, even when symptoms such as dry skin, sinus infections, rashes and nose bleeds continued for months or years, because they believed they were caused by the weather and would resolve with warmer seasons or climates:

The Boston winter made my skin dry out. And then I just started getting more [red patches] each winter. And so those things I noticed, but I never thought to bring them up to the doctor.* [Participant 30, diagnosed at 28, lymphoma]*

For a few female participants, beliefs or lack of knowledge about reproductive physiology influenced their interpretation of symptoms. One young woman thought it was common for people to grow lumps or feel pain during their menstrual cycle. Another had unexpected bleeding, but perceived there was considerable variability in normal reproductive functioning: “I just kind of, felt like maybe my body was built differently.” *[Participant 17, diagnosed at 35, cervical cancer].*

*Theme 3: Many young adults believed bodily changes to be low threat, based on perceived low susceptibility for serious diseases or lack of knowledge about cancer symptoms.* These participants explained characteristics they believed protected them from serious diseases such as cancer, including their age, health status, lack of family history, and practicing healthy behaviors: “I assumed I’m a healthy guy, [it’s just] a bug.” *[Participant 4, diagnosed at 28, leukemia]* A few described not suspecting cancer because they perceived themselves engaging in healthy behaviors including not smoking, drinking alcohol or tanning, or eating a healthy diet and exercising regularly:

Well, especially about skin cancer, I expected it to be like, sun related. Like, I’ve never tanned. I’m young, I’m healthy, I’m like, a runner, I love to exercise, I’m not eating fast food, I don’t tan, surely it’s not a problem. *[Participant 11, diagnosed at 37, melanoma]*

Some participants believed symptoms were low threat due to insufficient cancer symptom knowledge, such as one participant who experienced bladder infections, but this did not align with their knowledge of what types of symptoms warrant care: “It wasn’t like I found a lump in my breast. You know what I mean?” *[Participant 25, diagnosed at 27, ovarian cancer]* Another dismissed a breast lump as nonserious due to not having a family history: “There’s not been any history in my family…I know a part of my mind did go to it being cancerous, but I felt like, ‘Nah, that’s… that’s not possible.’ So I just like, waved it off.”* [Participant 6, diagnosed at 30, breast cancer].*

*Theme 4: Most participants sought information online about their symptoms as a method for avoiding professional medical evaluation while managing their low tolerance for uncertainty.* Participants who sought online information to help them self-appraise and self-manage symptoms described a long appraisal interval. Many recall conducting an online information search using the using Google search engine to look for information on their symptoms, e.g., “blood when passing gas*” [Participant 24, diagnosed at 39, colorectal cancer]* and adding in personal attributes “lumps on back, female 30 s.” *[Participant 11, diagnosed at 37, melanoma*] Others searched for specific causes of their symptoms e.g., “What would cause inflammation in your throat?’” *[Participant 30, diagnosed at 28, lymphoma]*. A few Googled symptom management tactics, such as, “How to deal with dry skin on your face.” *[Participant 30, diagnosed at 28, lymphoma]*.

Many participants who performed a Google search found information that they feel reduced their urgency for seeking care by offering a nonserious explanation or a method for alleviating symptoms. Some were guided to websites with at-home symptom management treatments. Others encountered webpages with an overwhelming and unhelpful list of potential causes— “It could be like, either tension headaches, or it could be a brain tumor.” [Participant 7, diagnosed at 27, brain cancer]— and were inclined to trust the less severe attributions: “Pretty much all I found was like, ‘This is a somewhat nonurgent issue.**’”** *[Participant 5, diagnosed at 20, sarcoma].*

*Theme 5: Young adults who did not experience a prolonged appraisal interval often had beneficial social network connections who implored seeking medical care.* Despite having low self-perceived urgency for seeking care, some participants experienced shorter appraisal intervals because they disclosed their symptoms to family, friends, and co-workers. A few young adults described an increase in their urgency for contacting a provider based on a desire to comfort or please loved ones who were pressing the need for evaluation: “[My wife] definitely kept pushing me to continue going to the doctor because I’ve never had anything like that wrong with me, you know?” *[Participant 9, diagnosed at 37, leukemia]* Other participants benefitted from social capital by receiving informal symptom evaluations from family members who worked the in medical field. For example, one participant’s mom worked at a cancer center:

I had also called my mom, because you know, she worked at cancer centers. And I was like, “Does this sound like something?” And yeah, she was like, mad at me [for not going to the doctor sooner], you know? *[Participant 27, diagnosed at 34, leukemia]*

## Research question #2: What prolongs the help-seeking interval (i.e., time from perceiving a need for medical evaluation to first consultation)?

*Theme 6: Most young adults did not experience delays within the help-seeking interval, but some delayed contacting a provider because they lacked health care access or had low confidence in healthcare***.** The majority of participants had the resources (e.g., insurance and a routine provider, e.g., primary care or OBGYN) to arrange a timely appointment once they perceived a need for medical consult. Several of these young adults described high self-efficacy for navigating the health system and access to social support as primary drivers of their prompt arranging of medical evaluation: “And like, as a 20-year-old I had scheduled [an appointment] by myself and had a friend take me. And so I had a lot of confidence about that.” *[Participant 25, diagnosed at 27, ovarian cancer]* Young adults without a routine provider sometimes sought timely medical care once perceiving a need; however, they typically presented directly to an emergency room or urgent care provider: “I had started to become a frequent flyer at an urgent care clinic.” *[Participant 3, diagnosed at 25, leukemia].*

Young adults who delayed contacting a provider often did not have adequate health care access and support for attending the appointment:

I should note that when the lump started showing up, I did not have health insurance. Not having health insurance, and with life being everything that it was, I was just like, I can’t even be bothered with this.* [Participant 11, diagnosed at 37, melanoma]*

Some had recently moved for work, school, or relationships and had not yet established primary care, while others were unable to miss work, such as one who was experiencing increasingly severe symptoms:

I had an intractable fever and chills, was not responding to Tylenol, was becoming more short of breath, and having more chest pain. But I did not want to go to the hospital because I just thought I was sick, and I couldn’t miss more work.* [Participant 1, diagnosed at 35, leukemia]*

Others described negative past healthcare experiences that instilled low confidence in medical care. One participant was hesitant to seek help because growing up in poverty and using Medicaid often led to feeling dismissed by doctors: “I did have those experiences with doctors not taking me very seriously, because I was poor and not worth their time,” *[Participant 22, diagnosed at 32, breast cancer]* Another avoided care because they felt every doctor attributed all symptoms to their weight status: “My experience had been—every time that I would go to a new doctor or try a new doctor, everything would come back to my weight.” *[Participant 15, diagnosed at 32, uterine]* One participant thought the risk of a dismissive encounter, like they had experienced previously, did not justify the financial cost of care: “If there’s a chance I’m going to be told it’s anxiety, why would I bother with a co-pay to be told this?” *[Participant 5, diagnosed at 20, sarcoma].*

## Research question #3: What prolongs the diagnostic interval (i.e., time from first medical consultation to formal diagnosis)?

*Theme 7: Many providers attributed symptoms to less serious causes or made no diagnosis, thus delaying young adults’ cancer diagnosis several months or more after their first medical consultation.* Participants who first presented at primary or urgent care often experienced significant delays during the diagnostic interval. Many young adults attended multiple appointments with several types of doctors, often receiving misdiagnoses or alternative symptom management suggestions before receiving the correct diagnosis. For example, one participant’s provider encouraged first trying a gluten-free diet to manage blood when passing gas*:* “Initially, they said that it might be what I was allergic to gluten, so they asked me to try stopping eating anything with gluten.”*[Participant 24, diagnosed at 39, colorectal cancer]* Several young adults expressed frustration at their provider not ordering medical testing for ongoing symptoms. One young adult explained: “I had to go to the doctor like, three to four times before she ordered the MRI.” *[Participant 7, diagnosed at 27, brain cancer]* Urgent care evaluations typically resulted in prescription medication or other at-home remedy suggestions. Several participants went to urgent care multiple times for the same symptoms, such as one with ongoing nonspecific illness: “They’d give me an antibiotic and basically say, ‘Go figure it out.’”* [Participant 3, diagnosed at 25, leukemia].*

Many young adults experienced a lack of follow-up testing or referrals to specialists and were forced to re-appraise whether they agreed with their providers’ less serious attributions or whether symptoms warranted seeking additional assessment. Most young adults trusted the information received at the initial appointment. For example, several young adults were told by a medical professional that their symptoms were most likely from stress, anxiety, depression, or lack of sleep, and they agreed with the plan to treat with medication, for example: “[The doctor said] ‘you’re probably fine, you’re sleep deprived. Here’s some Unisom, go home and get some sleep. And I’m like, “Okay.” *[Participant 18, diagnosed at 23, brain cancer].*

Conversely, some who did not trust the professional assessment they had received used online information seeking to find alternative explanations for symptoms: “Like, when the doctor told me it was lipomas, I looked up lipomas, and I like, read what they are. And I was like, “That doesn’t sound right, but like, he’s a doctor.” *[Participant 11, diagnosed at 37, melanoma]* After feeling brushed off or dismissed, participants’ self-advocacy abilities primarily drove their ability to seek additional care, as one participant explained:

I didn’t feel like the doctors really believed me, and I had to advocate for myself pretty hard. I walked through, you know, several different doctors escalating up to the ultimate biopsy. But each doctor I would hear the same thing. I would hear, “You’re too young. The lump is too near the surface, and you don’t have any family history....” *[Participant 22, diagnosed at 32, breast cancer]*

*Theme 8: The diagnostic interval was shorter when young adults’ initial provider encounter resulted in timely bloodwork, scans, or other tests; this was most common in emergency room evaluations.* Only a few young adults had primary or routine (e.g., OBGYN) providers who ordered bloodwork or other clinical tests at the first appointment: “My general practitioner ordered an ultrasound and referred me to a urologist.” *[Participant 2, diagnosed at 26, testicular cancer]* One participant who benefitted from prompt clinical testing had shared their wearable device data at the appointment: “Once I let my doctor know about [my high FitBit heart rate] she ordered an EKG. And then I got blood work done the next day and they also wanted to do a heart monitor.” *[Participant 20, diagnosed at 22, leukemia].*

Most participants who were diagnosed promptly after medical evaluation were those who presented directly to the emergency room with severe symptoms; the emergency room providers ordered bloodwork and CT scans in nearly all cases: “They gave me a CT scan, they did a blood panel.” *[Participant 8, diagnosed at 26, leukemia].*

## Discussion

Guided by the Model of Pathways to Treatment, we identified factors contributing to delayed cancer diagnosis among young adults across the appraisal, help-seeking, and diagnostic intervals, highlighting potentially modifiable targets for intervention. Most young adults experienced prolonged appraisal intervals due to misattributing symptoms to less serious causes. The help-seeking interval was relatively prompt for most participants, but delays occurred for some young adults who did not have adequate access and resources for seeking care, or who had low confidence in the care they would receive. Many young adults described lengthy delays in the diagnostic interval, often stemming from medical providers dismissing symptoms as nonserious. Across all intervals, young adults described a variety of delay factors that could be targeted in future intervention development.

### The appraisal interval

The misattribution of symptoms to external stressors drove appraisal interval delays when young adults believed it was normal to experience stress-related detrimental physical changes during certain life events. In a qualitative study of older adult cancer survivors’ symptom appraisal, attributions were not commonly to stress [54], suggesting this may be more common among young adults.

As in previous studies, in middle-aged and older adults [55, 56] as well as young adults [35, 57], insufficient cancer symptom knowledge was a consistent barrier to timely diagnoses. Insufficient knowledge of health may have contributed to participants perceiving symptoms as unserious (e.g., poor understanding of women’s health). Education about the types of bodily changes warranting medical evaluation is critical because, although symptoms such as lumps and bleeding can be benign, they are also associated with a variety of health issues and necessitate evaluation by a medical professional.

Several young adults delayed care seeking due to low perceived susceptibility to serious disease, including cancer. Some believed their internal characteristics, particularly young age and healthy behaviors, fully protected them from severe disease. This sense of protection based on age and health behaviors was not identified as a delay factor in a systematic review of middle-aged and older adult delays [56]. Lifestyle change-related campaigns targeting smoking [58] or tanning [59] may have had an unintended consequence of exaggerating people’s perceptions of the influence of health behaviors on illness risk.

A common response to symptom information insufficiency was to seek information about symptoms online. In most instances, this reduced urgency for medical evaluation, while some sought information after a provider’s dismissal of symptoms as nonserious. The role of online information seeking on diagnosis delays is uncertain. In a study of adults of all ages, those who sought online information about symptoms experienced longer delays between their first symptom search and their eventual disease diagnosis [60]. Conversely, online health information may help empower patients and improve their self-advocacy abilities [61, 62]. Given the greater use of online health information by young adults compared to older adults [41], it will be important to explore the association between cancer diagnosis delays and online information seeking in the younger demographic specifically.

### The help-seeking interval

Most young adults did not experience delays in the help-seeking interval; once they interpreted symptoms as warranting medical evaluation, they promptly arranged and attended a consultation. However, the young adults in this study had a higher rate of health care coverage than the larger US young adult population, and lack of health insurance is a crucial driver of young adult cancer diagnosis delays [30]. Some who felt they needed medical evaluation, but delayed pursuing a consultation, faced structurally rooted barriers to seeking care such as inability to miss work or negative past experiences with providers due to low socioeconomic status.

### The diagnostic interval

Most young adults expressed frustration with the delays they experienced within the diagnostic interval. Because young adult cancer is rare and presents with wide-ranging and often nonspecific symptoms, most providers are unlikely to suspect cancer in this age group. Some young adults with strong self-advocacy abilities continued pursuing additional appointments, while less self-confident participants felt resigned to trusting the doctor’s initial interpretation; the latter resulted in longer diagnostic intervals. Although providers may be limited by insurance coverage, increasing pressure to meet with more patients, and other constraints, the experiences patients have with timely emergency rooms diagnoses could serve as an example that ordering diagnostic testing has the potential to shorten disease diagnostic timeliness.

### Limitations and strengths

This study has several limitations. The sample is not statistically representative of US young adult demographics and was susceptible to selection bias that could limit our ability to understand the experiences of young adults who are less prone to participate in online research. There is also survival bias, as the experiences of patients who died from their disease or who have too severe symptoms to participate were not captured. Recall bias is possible given the study’s retrospective design and those interviewed closer to their diagnosis may have had better recall, but we are unable to evaluate accuracy. However, people often remember events surrounding significant life occurrences such as a cancer diagnosis [63, 64] and many patients in the study recalled extremely specific levels of detail about their symptoms (exact dates, exact google searches, weather on the day of appointments, etc.). We selected the current age eligibility cap of 45 pragmatically rather than on empirical grounds, and future studies should consider whether a narrower eligibility window better optimizes recall of pre-diagnosis experiences or whether a wider age range could improve recruitment of an already difficult-to-engage population. Related, we did not have eligibility criteria for time since diagnosis, and future studies should consider including explicit thresholds for this as well, as proximity to diagnosis may meaningfully influence recall quality. We are also unable to determine if a recalled symptom was related to the eventual cancer diagnosis; however, participants were probed to confirm if a provider concluded the relation between the symptom and disease was likely. A strength of this study is that it examined young adult cancer experiences across cancer types. While other studies have examined delays for specific types of cancer, such as early onset colorectal [65], there is a lack of research about general cancer delay causes for this age group. This study contributed needed insight into a growing disease burden in young adults. Finally, this study allows for comparison with other young adults cancer diagnostic pathway research by using constructs and definitions recommended by The Aarhus Statement [66].

### Implications for communication and future directions

Our findings suggest multiple opportunities for communication and education efforts aimed at shortening the time to diagnosis for young adult cancer. Even if young adults with symptoms do not receive a cancer diagnosis, timely care seeking should still be encouraged for certain symptoms that may also be indicative of other types of serious diseases. To improve the appraisal interval duration, symptom awareness messaging on commonly used digital sources could educate about young adult cancer symptoms, such as those listed in the CAUTION! model, a mnemonic list of seven symptoms and seven sites typical in young adult cancer that has successfully improved knowledge in high school students [32, 38].

Improving patient-provider communication is likely a key path for shortening young adult cancer diagnostic delays. Young people could be trained on self-advocacy and how to confidently navigate the healthcare system. Although most young adults with symptoms will not be diagnosed with cancer, this age group is experiencing a growing burden of multiple chronic diseases (e.g., hypertension, chronic liver disease, autoimmunity, cancer) [67–70]. Providers may be delaying accurate care and treatment for many conditions by not ruling out these more serious illnesses with diagnostic testing.

## Conclusion

Most young adults with cancer initially misattributed symptoms to stress, lifestyle, or minor issues, and thus delayed seeking medical evaluation. Many used online information to inform care-seeking decisions but encountered content that minimized symptom severity or offered unclear guidance. Upon seeking care, several young adults had to self-advocate for diagnostic testing after providers initially dismissed their concerns. Strategies to improve young adults’ awareness of potential cancer symptoms, address misconceptions about cancer risk, and promote timely care-seeking are warranted. Efforts should also focus on improving the quality of online symptom information and equipping young adults with tools to communicate more effectively with providers. While systemic changes are also needed, individual-level communication campaigns could help shorten time to diagnosis and improve outcomes for young adults with cancer.

## Data Availability

Qualitative interview data are not publicly available due to the potential for participant identification but may be available from the corresponding author on reasonable request and with appropriate ethical approvals.
